# Proteins and peptides in parotid saliva of irradiated patients compared to that of healthy controls using SELDI-TOF-MS

**DOI:** 10.1186/s13104-015-1641-7

**Published:** 2015-11-03

**Authors:** Alexa M. G. A. Laheij, Coen N. Rasch, Bernd W. Brandt, Johannes J. de Soet, Raymond G. Schipper, Arnoud Loof, Erika Silletti, Cor van Loveren

**Affiliations:** Department of Preventive Dentistry, Academic Centre for Dentistry Amsterdam, University of Amsterdam and VU University Amsterdam, Gustav Mahlerlaan 3004, 1081 LA Amsterdam, The Netherlands; Department of Radiation Oncology, The Netherlands Cancer Institute, Antoni van Leeuwenhoek Hospital, Plesmanlaan 121, 1066 CX Amsterdam, The Netherlands; Top Institute Food and Nutrition, PO Box 557, 6700 AN Wageningen, The Netherlands; Central Laboratory for Haematology, Radboud University Nijmegen Medical Centre Post 476, PO Box 9101, 6500 HB Nijmegen, The Netherlands; NIZO Food Research B.V., P.O. Box 20, 6710 BA Ede, The Netherlands

**Keywords:** Head and neck cancer, Saliva, Proteomics, Parotid gland, SELDI-TOF-MS

## Abstract

**Background:**

Radiotherapy to the head and neck area damages the salivary glands. As a consequence hyposalivation may occur, but also the protein composition of saliva may be affected possibly compromising oral health. The aim of our study was to compare the relative abundance of proteins and peptides in parotid saliva of irradiated patients to that of healthy controls.

**Methods:**

Using Lashley cups and citric acid, saliva from the parotid glands was collected from nine irradiated patients and ten healthy controls. The samples were analyzed with SELDI-TOF-MS using a NP20 and IMAC-30 chip in the molecular weight range of 1–30 kDa.

**Results:**

On the NP20 chip 61 (out of 217) and on the IMAC-30 chip 32 (out of 218) peaks differed significantly in intensity between the saliva of the irradiated patients and healthy controls. 55 % of the significant peaks showed higher intensity and 45 % showed lower intensity in the saliva of irradiated patients. The peaks may represent, amongst others, the salivary proteins lysozyme, histatins, cystatin, protein S100 and PRP’s.

**Conclusions:**

Large differences were found in the relative abundance of a wide range of proteins and peptides in the parotid saliva of irradiated patients compared to healthy controls.

**Electronic supplementary material:**

The online version of this article (doi:10.1186/s13104-015-1641-7) contains supplementary material, which is available to authorized users.

## Background

Radiotherapy is often needed for the management of a tumor in the head and neck area. Usually radiotherapy involves the primary treatment of a tumor, but it can also be used additionally to surgery or as part of palliative care. The radiation-induced consequences for the healthy oral tissues can be divided into early and late effects. The early effects consist of damage to the oral mucosa, salivary glands and taste. Late effects comprise damage to the salivary glands, dentition, periodontium, bone, muscles and the joints [1, 2].

Damage to the salivary glands leads to a rapid decrease in salivary flow after the start of radiotherapy [3]. Weeks after the start of radiotherapy, the flow rate gradually starts to recover and only after 5 years the mean parotid flow rate may return to baseline levels [4, 5]. However, 21 % of patients still suffered from a significantly lowered salivary flow 5 years after radiotherapy [5].

There are secondary effects related to changes in salivary flow and to changes in composition. Saliva is an important host defense mechanism helping to keep the oral cavity free of diseases like caries, gingivitis, periodontitis and infections as a result of antimicrobial, buffering and remineralizing capacities [6, 7]. There is a risk for radiation caries, a form of caries that develops very rapidly when salivary functions are disturbed and affects tooth surfaces that are normally resistant to the development of caries [8].

Also the changes in the composition of saliva imply that the salivary pH and buffer capacity, as a result of a lower bicarbonate concentration, are significantly lower compared to healthy controls [9, 10] and the concentrations of sodium, chloride, calcium, potassium, phosphate and magnesium in saliva have been changed [9–12]. The oral microflora also changes in composition. The increase of the acidogenic bacteria *Streptococcus mutans* and *Lactobacillus* species and the yeast *Candida* may be a further threat for dental health [13–16].

After radiotherapy the total protein concentration may increase [9–11]. For individual proteins, it was found that the concentration of acidic proline-rich proteins (PRP’s), amylase, epidermal growth factor and MUC5B in the saliva of irradiated patients was lower than in healthy controls [9, 17–19], whereas the levels of lactoferrin, lysozyme, albumin, peroxidase, IgA, IgG and haptocorrin in saliva were elevated after irradiation [9–12, 18, 20]. When interpreting the changed concentrations of salivary proteins, it should be taken into account that the amount of salivary flow determines the total availability of these proteins in the oral cavity [10, 21].

The salivary proteins studied in irradiated patients so far make up only a small proportion of the total amount of proteins and peptides in saliva [6, 22]. More knowledge on the changes in a wide range of salivary proteins and peptides could make it possible to screen patients for specific markers for the prediction of oral health, to monitor effects of treatment or preventive measures and to follow the composition over time.

Surface enhanced laser desorption/ionization time-of-flight mass spectrometry (SELDI-TOF-MS) is a method for the proteomic analysis of saliva [23]. It is a high throughput method that produces protein/peptide profiles of biological samples in the range of about 1–30 kDa [24, 25]. This technique separates proteins and peptides using their chemical and physical characteristics. Upon laser desorption and ionization an accurate protein/peptide profile of the sample is generated. SELDI-TOF-MS was used in several clinical studies to discover biomarkers [26–30] in saliva and to examine proteomic profiles in the saliva of hematopoietic stem cell transplant patients [31].

Our hypothesis was that many more salivary proteins differ between irradiated patients and healthy controls than those that were studied before. We conducted a cross-sectional study in which we compared proteins and peptides in parotid saliva between healthy controls and patients that were irradiated in the head and neck area. The aim of our study was to make an inventory of the differences in the relative abundance of a wide range of proteins in the parotid saliva of irradiated patients and healthy controls using SELDI-TOF-MS.

## Methods

### Subjects

Nine patients (5 females, 4 males) who had received radiotherapy for a tumor in the head and neck area participated in this study. The mean age of the patients was 60.7 (±11.9) years old. Radiotherapy took place over 6 months ago. In all patients the parotid glands were in the field of irradiation. The mean dosage of irradiation on the parotid glands was 29.3 (±11.5) Gy.

Ten healthy volunteers (7 males, 3 females) also participated in this study. Their mean age was 39.8 (±12.2) years old. They had no known abnormalities in their salivary glands and they did not use any medication affecting salivary flow.

The medical ethical committee of the NKI/AvL hospital was consulted about this study. The medical ethical committee determined that this study was exempt from ethics approval since the study was about the non-invasive collection of a single sample in a small group of patients. All participants gave informed consent.

### Saliva collection and storage

Saliva from the left and right parotid gland was collected using Lashley cups. Salivary flow was stimulated by applying a 4 % citric acid solution to the lateral border of the tongue every 30 s. Saliva was collected during 4 min. After collection a proteinase inhibitor cocktail (PIC; P8340, Sigma, St. Louis, MI, USA) was added to the samples. The samples were frozen immediately in liquid nitrogen and kept at −80 °C until analysis.

### Saliva handling and analysis

The saliva samples were analyzed using SELDI-TOF-MS (Bio-Rad, Freemont, USA). Two ProteinChip surfaces were used: the IMAC-30 chip (binds proteins based on metal affinity) and the NP20 chip (general binding of proteins). All chips were loaded according to the manufacturer’s instructions (Bio-Rad).

Saliva samples were thawed and centrifuged at 10,000*g* for 5 min. The supernatants were used for analysis. The NP20 chip preparation procedure started with washing of each spot with 5 µl PBS. The PBS was removed and 5 µl of sample per spot was loaded. The chips were incubated at room temperature for 20 min under gentle agitation. The chips were washed twice with PBS and air-dried at room temperature for 15 min. Matrix sinapinic acid (50 % ACN, 0.5 % TFA) was applied twice (0.8 µl each time and 1 min apart). Finally, the chips were air-dried at room temperature.

The IMAC-30 chip preparation procedure started with loading of 50 µl of 0.1 M copper sulphate per spot for 10 min. The spots were washed with 150 µl demineralized water. Then the chip surface was neutralized using 150 µl 0.1 M sodium acetate buffer at pH 4.0 per spot for 5 min. After washing with 150 µl demineralized water the spots were pre-incubated twice with 150 µl of binding buffer (0.1 M Tris–HCL, pH 7.4) for 5 min. 1 µl of sample and 99 µl of binding buffer per spot were incubated for 1 h at room temperature under agitation. The spots were washed twice with 150 µl binding buffer for 5 min, once with demineralized water and air-dried. Matrix sinapinic acid (50 % ACN, 0.5 % TFA) was applied twice (0.8 µl each time and 1 min apart) and air-dried.

The externally calibrated chips were read on a ProteinChip Reader IIC instrument (Bio-Rad). The following settings were used: detector voltage 2500 V, acquired mass range from 1 to 30 kDa, focus mass 15 kDa, matrix attenuation was 1 kDa sampling rate at 400 MHz, 2 warning shots were fired at each position, which were not included in the collection, 5 data shots were fired at 2500 nJ.

### Protein prediction

From SELDI-TOF-MS data it is not possible to exactly determine to which (part of) specific proteins and peptides the found peaks correspond as a result of a low mass resolution and a lack of tandem mass spectrometric capabilities, which limits the identification of proteins and peptides [25].

The TagIdent tool (http://web.expasy.org/tagident/) was used to search for proteins that matched the significant SELDI peaks using Swiss-Prot as the database, with human as the organism, with and without the keyword ‘secreted’. Searches were carried out using the molecular weight data obtained from the SELDI-TOF-MS analysis with a 1 % molecular weight range.

### Statistical analysis

Peak labeling and statistical analyses of the SELDI-TOF-MS data were performed with ProteinChip Data Manager Software (version 3.0) for peaks with a signal-to-noise ratio of ≥3.0 in the range from 1 to 30 kDa. Normalization of the data did not influence overall results. Non-parametric Mann–Whitney tests were performed to compare protein profiles between groups. For the correction of multiple testing, the FDR method was used [32], with a critical value for the false discovery rate of 0.15 and a total number of tests of 435. That corresponded to a p value <0.01. Other statistical analyses were carried out using the statistical software package SPSS version 18.

## Results

**Fig. 1:**
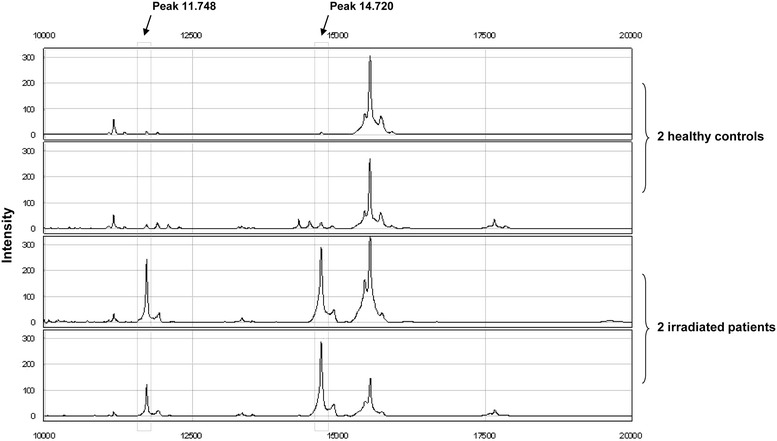
SELDI-TOF-MS protein profiles of saliva samples. Protein profiles of saliva samples of two irradiated patients and two healthy controls obtained with the IMAC-30 chip

Figure 1 depicts a protein profile of two patients and two healthy controls generated by the IMAC-30 chip. This figure displays amongst others peaks with an *m/z* value of 11,748 and 14,720. These peaks were present in all samples, but they were clearly higher in intensity in the saliva samples of the irradiated patients. Other peaks in this figure were present in all samples and do not differ significantly in intensity between the irradiated patient group and the healthy controls.

On the NP20 chip in total 217 unique peaks were detected. 29 Peaks showed a significant higher intensity and 32 peaks showed a significant lower intensity in the saliva of the irradiated patients (Table 1). All significant peaks are shown in Additional file 1: S1.

**Table 1:** Distribution of peaks that differed significantly in intensity on the NP20 chip

*m/z* ratio	Number of peaks	Higher in intensity in patients	Lower in intensity in patients
0–5000	27	6	21
5000–10,000	11	4	7
10,000–15,000	13	10	3
15,000–30,000	10	9	1
Total	61	29	32

On the IMAC-30 chip in total 218 peaks were detected. 23 Peaks showed a significant higher intensity and 9 peaks showed a significant lower intensity in the irradiated patients (Table 2). All significant peaks are shown in Additional file 1: S1.

**Table 2:** Distribution of peaks that differed significantly in intensity on the IMAC-30 chip

*m/z* ratio	Number of peaks	Higher in intensity in patients	Lower in intensity in patients
0–5000	7	2	5
5000–10,000	4	4	0
10,000–15,000	12	10	2
15,000–30,000	9	7	2
Total	32	23	9

When comparing the saliva samples from the left and right parotid glands no significant differences were found in peak intensity, neither for the healthy controls, nor for the irradiated patients. The amount of irradiation that the patients received on their left and right parotid glands did not differ significantly (p = 0.866).

The *m/z* values of all the 93 significantly different peaks were entered in the TagIdent tool and the search resulted in 3528 unique human Swiss-Prot entries. These Swiss-Prot entries were intersected with a list of 917 known salivary proteins and peptides with International Protein Index (IPI) identifiers (20). The IPI identifiers were mapped to Swiss-Prot using the ID-mapping tool of UniProtKB (http://www.uniprot.org). 143 IPI identifiers could not be mapped. The intersection resulted in 186 unique predicted proteins of which 65 are secreted, according to Swiss-Prot. Table 3 lists these 65 predicted proteins of which some have functions that are beneficial to oral health like lysozyme, histatins, proline rich proteins (PRP’s) and S100 proteins.

**Table 3:** Secreted proteins and peptides that resulted from the database search

UniProt accession	Protein name
P31947	14-3-3 protein sigma
P01011	Alpha-1-antichymotrypsin
P03973	Antileukoproteinase
P04280	Basic salivary proline-rich protein 1
P10163	Basic salivary proline-rich protein 4
P61769	Beta-2-microglobulin
P13688	Carcinoembryonic antigen-related cell adhesion molecule 1
P13987	CD59 glycoprotein
P10909	Clusterin
P01034	Cystatin-C
P09228	Cystatin-SA
P01037	Cystatin-SN
P81605	Dermcidin
P19957	Elafin
Q16610	Extracellular matrix protein 1
P08294	Extracellular superoxide dismutase [Cu–Zn]
P02751	Fibronectin
Q8NFU4	Follicular dendritic cell secreted peptide
P47929	Galectin-7
P28799	Granulins
P00738	Haptoglobin
P15515	Histatin-1
P15516	Histatin-3
P01591	Immunoglobulin J chain
P15814	Immunoglobulin lambda-like polypeptide 1
Q14624	Inter-alpha-trypsin inhibitor heavy chain H4
P18510	Interleukin-1 receptor antagonist protein
Q9NZH8	Interleukin-36 gamma
Q9UBX7	Kallikrein-11
Q9UKR0	Kallikrein-12
Q92876	Kallikrein-6
P49862	Kallikrein-7
P02788	Lactotransferrin
P61626	Lysozyme C
P09237	Matrilysin
Q13421	Mesothelin
O95467	Neuroendocrine secretory protein 55
P59665	Neutrophil defensin 1
P80188	Neutrophil gelatinase-associated lipocalin
O75594	Peptidoglycan recognition protein 1
P00747	Plasminogen
Q16378	Proline-rich protein 4
O75629	Protein CREG1
P58499	Protein FAM3B
P31151	Protein S100-A7
P05109	Protein S100-A8
P06702	Protein S100-A9
P00734	Prothrombin
P02753	Retinol-binding protein 4
P07998	Ribonuclease pancreatic
O00584	Ribonuclease T2
P02810	Salivary acidic proline-rich phosphoprotein 1/2
P36952	Serpin B5
Q99954	Submaxillary gland androgen-regulated protein 3A
P02814	Submaxillary gland androgen-regulated protein 3B
P10599	Thioredoxin
O60235	Transmembrane protease serine 11D
P07477	Trypsin-1
P35030	Trypsin-3
Q6MZM9	Uncharacterized protein C4orf40
P11684	Uteroglobin
P04004	Vitronectin
Q6PCB0	von Willebrand factor A domain-containing protein 1
Q14508	WAP four-disulfide core domain protein 2
Q96DA0	Zymogen granule protein 16 homolog B

## Discussion

In this study, the rapid and high throughput SELDI-TOF-MS technique was successfully used to show differences in proteins and peptides in the saliva of irradiated patients compared to healthy controls. The results are in agreement with our hypothesis; many more proteins were found to differ in intensity between the two groups than were studied previously. With this method, it is not only possible to compare protein profiles between groups, but also to rapidly screen patients for a specific protein marker related to (oral) disease and health, to observe the effects of a treatment or preventive measures and to follow composition in time.

Large differences in the composition of parotid saliva between irradiated patients and healthy controls were found: 93 peaks, measured by SELDI-TOF-MS, differed significantly in intensity between both groups. Significant peaks can correspond to known proteins and peptides like lysozyme, histatins, PRP’s and S100 proteins. No differences were found in the protein composition of parotid saliva between the left and right parotid gland of both healthy controls and irradiated patients. This is in agreement with a previous study [25].

Until now, most studies were targeted at specific proteins in the saliva between irradiated patients and healthy controls or before and after radiation treatment [9, 10, 17, 33]. Differences were reported in lysozyme and PRP’s concentrations, predicted proteins that are found in our study as well [10, 17, 19]. The interesting point of our study is that we looked at a wide range of proteins and peptides identifying differences that were unnoticed until now. Some of the differences may comprise potential key proteins and peptides in determining oral health.

Other studies with SELDI-TOF-MS found significant differences in peaks between diseased and healthy persons that correspond to our results [26, 28, 31]. Shintani et al. [26] identified a marker for oral squamous cell carcinoma that consisted of truncated cystatin SA. Ryu et al. [28] found several different peaks in Sjögren patients and identified two of the peaks as lysozyme C and cystatin C. Imanguli et al. [31] studied salivary changes in hematopoietic stem cell transplant patients. They found 78 significantly different peaks comparing pre- and post transplant samples. One peak was identified as cystatin SN. Future studies have to reveal whether various diseases and treatments bring about comparable changes in the salivary protein composition.

We searched for candidate proteins corresponding to the peaks with various protein databases. Among the proteins listed in Table 3 are proteins known to protect oral health, of others the importance is not clear yet. Lysozyme is an enzyme that kills bacteria by breaking down their cell wall. Cystatins are protease-inhibitors that contribute to the prevention of oral infections. The synthesis of cystatins is elevated, when the protective mechanisms in the mouth fail and oral infections originate [34]. Histatins are peptides with an antimicrobial function [35, 36]. Furthermore, histatins exhibit wound healing properties as well [37]. Calprotectin (protein S100A8 and A9) is a zinc- and calcium-binding protein that exhibits antimicrobial activity. It exhibits bacteriostatic properties and is an inflammatory marker in serum [38]. The levels of calprotectin in saliva are higher in the case of oral infections [39]. PRP’s are involved in the adherence of bacteria to the enamel pellicle [6]. This study indicates that oral health may be associated with changes in the whole salivary proteome, rather than only a few proteins, Therefore we need to study the whole salivary proteome to find associations and possible mechanisms between these proteins and oral health.

The mean age of the patients in our study was higher than of the healthy controls, due to one older patient. The changes in protein composition due to age are minimal and usually related to age-related medical conditions, as in our study [40]. In addition, by correcting the p value for multiple testing, we only found large differences in the relative abundance of proteins in parotid saliva. Therefore, these differences have to be related to the impaired health condition and not to age differences.

The specific cause of the changes in protein composition that we found is not clear. It is possible that the disease itself altered the composition of saliva, but it can also be the (amount of) irradiation that caused the changes. To distinguish between these possible options, it is necessary to compare the salivary protein composition of the cancer patients before and after radiotherapy.

In conclusion, many differences are found in the protein and peptide composition of parotid saliva of patients irradiated in the head and neck area compared to healthy controls. Of some proteins the relation with oral health is known, for others it remains to be established. It is important to look at a broad range of proteins to identify associations between changes in the salivary proteome and health or disease that were unnoticed until now. Further research is needed to prospectively study changes in salivary proteins and to relate those changes to oral health.

## Additional file

10.1186/s13104-015-1641-7 All significant peaks (m/z ratio) with corresponding p-values.
