# Supplementary material for: Proteins and peptides in parotid saliva of irradiated patients compared to that of healthy controls using SELDI-TOF-MS
Source: BMC Res Notes. 2015 Nov 3;8:639. doi: 10.1186/s13104-015-1641-7 (PMC4632372; doi:10.1186/s13104-015-1641-7)
Supplement: Supplementary file 1 — 10.1186/s13104-015-1641-7 All significant peaks (m/z ratio) with corresponding p-values. [file 13104_2015_1641_MOESM1_ESM.doc]

**Additional** file S1. All significant peaks (m/z ratio) with corresponding p-values

| IMAC chip | | NP20 chip | |
| --- | --- | --- | --- |
| Peaks (m/z ratio) | p-value | Peaks (m/z ratio) | p-value |
| 2,997 | 0,0038 | 1,068 | 0,0001 |
| 3,283 | 0,0031 | 1,191 | 0,0042 |
| 3,410 | 0,0021 | 1,212 | 0,0009 |
| 3,778 | 0,0019 | 1,234 | 0,0038 |
| 4,102 | 0,0031 | 1,257 | 0,0020 |
| 4,320 | 0,0056 | 1,279 | 0,0026 |
| 4,989 | 0,0061 | 1,481 | 0,0007 |
| 7,358 | 0,0004 | 1,507 | 0,0015 |
| 7,464 | 0,0034 | 1,756 | 0,0003 |
| 9,801 | 0,0088 | 2,513 | 0,0002 |
| 9,872 | 0,0019 | 2,789 | 0,0001 |
| 11,366 | 0,0001 | 2,816 | 0,0000 |
| 11,394 | 0,0073 | 2,878 | 0,0003 |
| 11,654 | 0,0000 | 2,972 | 0,0000 |
| 11,692 | 0,0015 | 3,004 | 0,0000 |
| 11,731 | 0,0010 | 3,067 | 0,0083 |
| 11,748 | 0,0001 | 3,156 | 0,0000 |
| 11,875 | 0,0012 | 3,19 | 0,0017 |
| 11,952 | 0,0001 | 3,259 | 0,0005 |
| 13,232 | 0,0042 | 3,451 | 0,0059 |
| 13,370 | 0,0096 | 3,846 | 0,0007 |
| 14,720 | 0,0003 | 4,008 | 0,0003 |
| 14,915 | 0,0005 | 4,381 | 0,0002 |
| 15,149 | 0,0001 | 4,437 | 0,0029 |
| 15,738 | 0,0031 | 4,503 | 0,0090 |
| 15,929 | 0,0001 | 4,614 | 0,0024 |
| 17,227 | 0,0073 | 4,686 | 0,0097 |
| 19,585 | 0,0046 | 5,486 | 0,0083 |
| 22,640 | 0,0073 | 5,573 | 0,0018 |
| 24,269 | 0,0019 | 5,757 | 0,0004 |
| 25,669 | 0,0067 | 5,949 | 0,0014 |
| 27,381 | 0,0003 | 6,126 | 0,0035 |
|  |  | 6,904 | 0,0017 |
|  |  | 6,972 | 0,0009 |
|  |  | 7,805 | 0,0097 |
|  |  | 7,889 | 0,0070 |
|  |  | 8,981 | 0,0064 |
|  |  | 9,673 | 0,0064 |
|  |  | 10,256 | 0,0001 |
|  |  | 10,475 | 0,0083 |
|  |  | 10,842 | 0,0029 |
|  |  | 11,105 | 0,0000 |
|  |  | 11,182 | 0,0001 |
|  |  | 11,2 | 0,0000 |
|  |  | 11,222 | 0,0000 |
|  |  | 11,245 | 0,0042 |
|  |  | 11,406 | 0,0020 |
|  |  | 11,513 | 0,0017 |
|  |  | 13,305 | 0,0001 |
|  |  | 13,353 | 0,0004 |
|  |  | 14,337 | 0,0064 |
|  |  | 15,315 | 0,0076 |
|  |  | 15,404 | 0,0000 |
|  |  | 15,465 | 0,0000 |
|  |  | 15,465 | 0,0000 |
|  |  | 15,561 | 0,0000 |
|  |  | 15,747 | 0,0000 |
|  |  | 15,925 | 0,0010 |
|  |  | 18,971 | 0,0005 |
|  |  | 20,991 | 0,0035 |
|  |  | 26,854 | 0,0001 |
